# Precipitation in July maximizes total above-ground productivity of the desert steppe in Inner Mongolia, China

**DOI:** 10.1371/journal.pone.0314983

**Published:** 2024-12-16

**Authors:** Chunxue Han, Ruichao Li, Haigang Li

**Affiliations:** 1 College of Resources and Environmental Sciences, Inner Mongolia Agricultural University, Hohhot, China; 2 Inner Mongolia Key Laboratory of Soil Quality and Nutrient Resource, Hohhot, China; 3 Key Laboratory of Agricultural Ecological Security and Green Development at Universities of Inner Mongolia Autonomous, Hohhot, China; Tennessee State University, UNITED STATES OF AMERICA

## Abstract

Precipitation distribution during the growing season and interannual precipitation variation may have significant impacts on grassland ecosystem productivity at the site level. To explore the effect of the distribution of precipitation on plant communities in the Inner Mongolian desert steppe dominated by *Stipa breviflora*, we analyzed monthly precipitation patterns during the growing season (May–October) over the past 60 years (1961–2020) and identified four major precipitation distribution patterns. These included the concentrated precipitation during July (TΛ7), August (TΛ8), and during the early and late growth stages. However, with precipitation being scarce during the boom (TM), the distribution resembled a normal distribution (T∩). Field experiments simulating the four distributions were conducted from May to October 2021. The results showed that the effects of the distribution of precipitation on plant species, diversity, and abundance were not significant; only the Pielou evenness showed a significant effect after July. The total above-ground net primary productivity (ANPP) of TΛ7 was 55.4% higher than those of the other three patterns, whereas the differences among the other three precipitation distributions were not significant. The annual forb *Neopallasia pectinate* was the primary contributor to the increased ANPP of TΛ7. These results suggest that the *S*. *breviflora* desert steppe achieved maximum productivity when the precipitation reached 41.6% of the annual average during July and satisfied the basic plant growth requirements during other months. This study emphasizes the implementation of management measures (irrigation or artificial precipitation) for maximizing forage yield and forecasting the plant composition in desert steppes.

## Introduction

The frequency of extreme precipitation events under global change has complicated the plant community structure and composition [1–6], thereby affecting ecosystem functioning [7, 8]. Predicting the impact of precipitation on biodiversity is relevant for global change models in water-limited ecosystems [9]. Moreover, precipitation is an important factor in the production of biomass, particularly in arid environments [10, 11]. Above-ground net primary productivity (ANPP) is a basic feature of all ecosystems and is closely related to material and energy cycles [12, 13]. The impact of rainfall on the productivity of desert steppes, for example, differs from that on grasslands, which are only slightly restricted by water [14]. The determination of the controlling factors of ANPP is an important goal of ecosystem ecology. Thus, an understanding of the effects of precipitation on biodiversity and productivity in desert steppe ecosystems is important in the face of global climate change.

In grassland ecosystems, most plants grow rapidly within a short period after precipitation events, and the frequency and intensity of precipitation affect annual productivity. This leads to a weak association between grassland ecosystem productivity and annual precipitation at the site level [15, 16]. However, this also leads to difficulty in extending the conclusions from single-point experiments to the entire biome [14]. Increasing evidence has indicated that the effect of the distribution of seasonal precipitation on productivity cannot be ignored and that it is as important as the total amount of precipitation regarding its effect on ANPP [1, 4, 17, 18]. Based on a 24-year study (1980–2003) of an Inner Mongolian grassland, Bai *et al*. [19] reported that January–July precipitation was the primary climatic factor causing fluctuations in community biomass, except in extraordinarily wet years. Peng *et al*. [20] reported that due to the hysteresis effect of precipitation and soil moisture, higher spring (May–June) precipitation increased soil moisture during spring and summer (July–August), leading to increased summer and annual grassland productivity in Inner Mongolia, and this could even offset the impact of summer decreases in precipitation on productivity. Accumulated precipitation during the non-growing season is beneficial to soil water retention, plant warming, and germination, factors that can increase the accumulation of biomass [21]. In addition, researchers have highlighted the importance of precipitation in grasslands during the previous year [4, 22–24]. The lag effect of rainfall on productivity depends on water availability, which is in turn determined by the sensitivity of climatic and soil factors to precipitation in drylands [25–27]. Desert steppes are vulnerable to prolonged drought and low soil water utilization efficiency [28, 29]. The response of ANPP to the distribution of precipitation may differ among ecosystems. Moreover, most such studies are based on long-term observations or modeling analyses, but manipulative experiments can more directly verify the relationship between precipitation distribution and ANPP.

Desert steppes, located in the transition zones between desert and grassland, are very sensitive to changes in precipitation [30]. Post and Knapp [31] found that a rainstorm during the early, middle, or late growing season stimulated basic ecosystem processes (i.e., above-ground net primary production and growth of the dominant plant species) in the semi-arid shortgrass steppe of North America. In the case of constant total precipitation, long-term increases in the variability of precipitation during the growing season stimulated the turnover of rare and uncommon species, thereby altering the composition of the plant community and indirectly leading to a decrease in ANPP [17]. In conclusion, the effects of changes in rainfall patterns during the growing season on the community composition and productivity of desert steppe ecosystems should not be ignored. Jobbágy and Sala [32] emphasized that distinguishing the responses of functional ANPP types to precipitation could accurately reveal the impact of climate change on grassland productivity. Therefore, the effects of the distribution of precipitation on arid ecosystems during the growing season should not be overlooked when predicting the responses to climate change. The purpose of this study was to evaluate the effects of the distribution of precipitation during the growing season on the plant community composition and above-ground productivity in a desert grassland ecosystem. Two hypotheses guided our study: (1) plant diversity would change along with the distribution of precipitation during the growing season, and (2) the distribution of precipitation would determine ANPP independent of the total amount of precipitation.

## Materials and methods

### Study area

**Fig 1:**
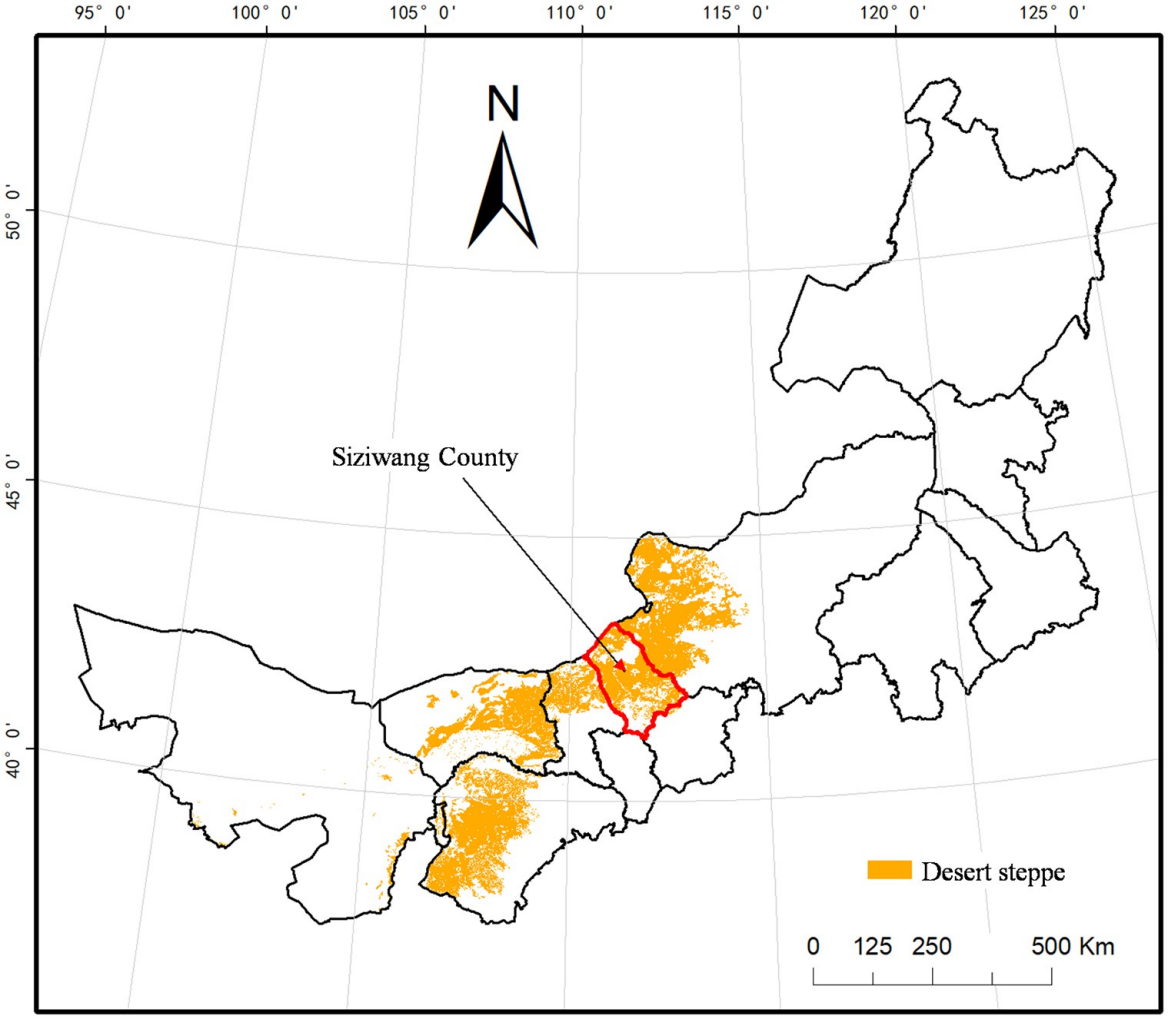
The map of desert steppe and the location of the study area.

This study was conducted in Adege, Siwangqi Banner, Ulanqab, Inner Mongolia Autonomous Region, China, an area located in a temperate arid and semi-arid continental monsoon climate region (Fig 1). The geographical coordinates of the study area are 112°30′57″ E, 42°02′17″ N, and the elevation is 1328 m. The annual average (1961–2020) precipitation is 304 mm and is concentrated from June to September and accounts for > 70% of the total annual precipitation, whereas the annual average water surface evaporation is 2180 mm [33]. The annual average air temperature is 3.7°C and is 14.3°C (12.9–16.5°C) during the growing season. The soil type is light chestnut, and the soil texture is sandy loam. The experimental site was located in a desert steppe dominated by *Stipa breviflora* Griseb, *Neopallasia pectinate* (Pall.) Poljak, and *Cleistogenes songorica* (Roshev.) Ohwi, with *Artemisia macilenta* (Maxim.) Krasch, *Artemisia frigida* Willd, and more than 10 other herbs in addition to shrubs such as *Caragana korshinskii* Kom. The study area was grazed from October to April and fenced from May to September when it was used as a winter pasture. Under the influence of climate change and human activity, desert grassland ecosystems have been severely damaged, with large-scale degradation, reduced species richness, a single vegetation type, and low grassland productivity [34, 35]. The basic soil and vegetation conditions in the study area are listed in Table 1.

**Table 1:** Soil physicochemical properties and vegetation community characteristics in the study area.

SOM (g/kg)	pH	Olsen-P (mg/kg)	NH_4_OAC exchangeable K (mg/kg)	NO_3_^-^-N (mg/kg)	NH_4_^+^-N (mg/kg)	Plant species	Aboveground biomass (g/m^3^)
18.88	8.5	1.19	208.63	1.17	1.05	14	66.86

### Experimental design

#### Selection of precipitation distributions

**Fig 2:**
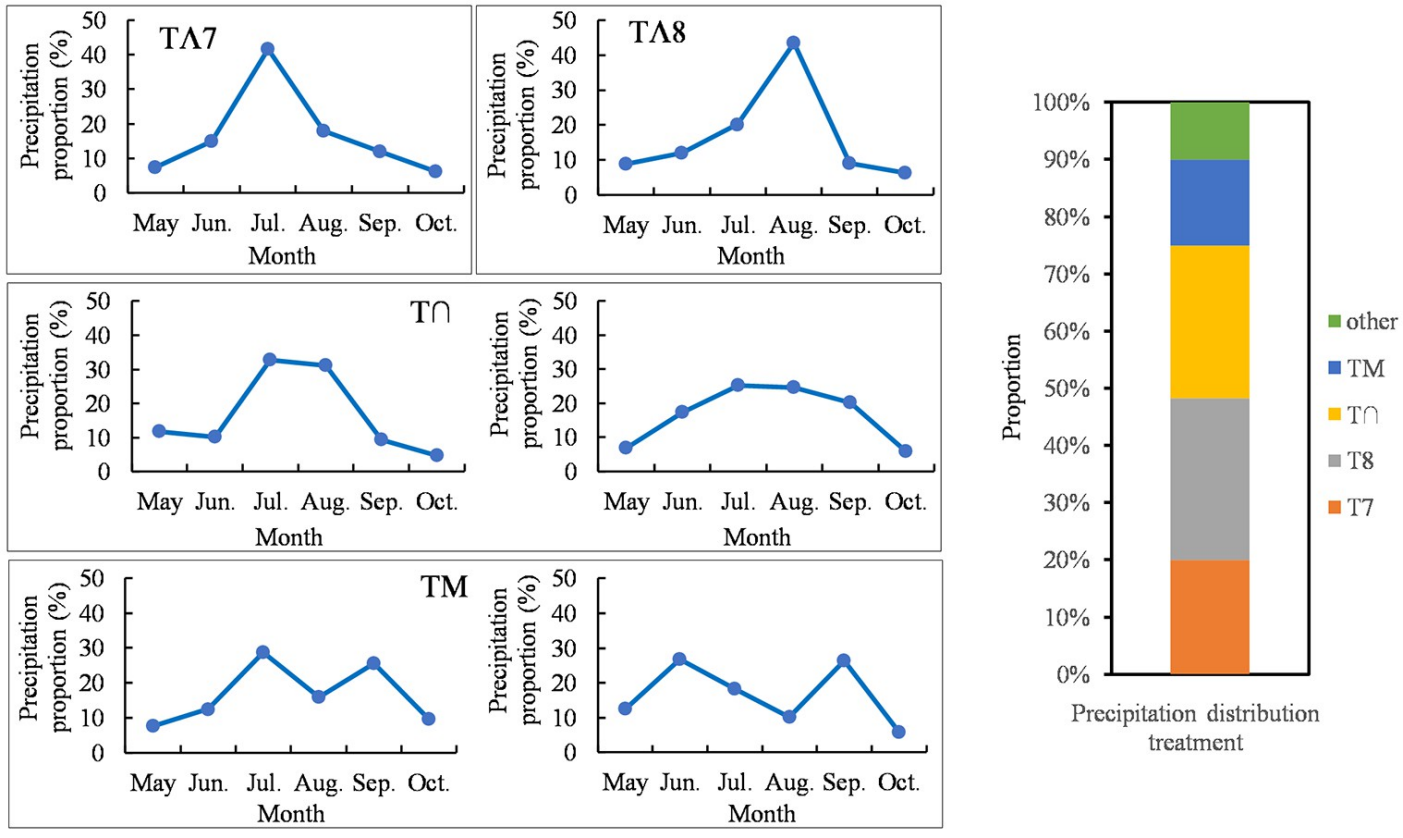
Four precipitation distribution patterns and the proportion of each pattern during the growing season (May–October) from 1961 to 2020.

Throughout the 20th century, studies have documented potential alterations in annual precipitation amount, alongside heightened fluctuations in precipitation patterns on both inter- and intra-annual scales [36–38]. In water-limited ecosystems, changes in soil moisture caused by changes in precipitation regimes affect aboveground net primary production [39]. Therefore, the precipitation data for the Siziwang Banner growing season (May–October) from 1961 to 2020 were used to classify the distribution of precipitation and were obtained from the China Meteorological Data Network (http://data.cma.cn/). By analyzing the distribution in the growing season of the *S*. *brevifloris* desert steppe, four main precipitation patterns were identified (Fig 2) covering 90% of the past 60 years. Concentrated precipitation occurred during July for 12 years and during August for 17 years, accounting for 20% and 28.3%, respectively, making this the primary distribution pattern. July and August are represented by TΛ7 and TΛ8, respectively. The distribution resembled a normal distribution during the growing season for 16 years, accounting for 26.7%; this is represented by T∩. The final pattern occurred when concentrated precipitation occurred during the early and late growth stages with little precipitation during the flourishing period in 9 years, accounting for 15%. This pattern is represented by TM. An annual average precipitation of 275.8 mm was taken as the total amount, and the monthly average precipitation events were counted as the watering events from May to October during 1961–2020. The watering events and amounts corresponding to the four precipitation distribution patterns are listed in Table 2.

**Table 2:** Monthly watering events, individual watering amounts, and monthly precipitation ratios from May to October corresponding to the four precipitation distribution patterns.

	Treatment	May	Jun.	Jul.	Aug.	Sep.	Oct.
Amount (mm)	TΛ7	5.1	8.2	19.1	8.3	6.6	4.3
	TΛ8	6.1	6.6	9.3	20.0	5.0	4.4
	T∩	4.7	9.5	11.6	11.3	11.2	4.0
	TΜ	8.6	14.7	8.4	4.7	14.6	4.0
Events	All	4	5	6	6	5	4
Ratio (%)	TΛ7	7.4	14.9	41.6	18.1	12.0	6.2
	TΛ8	8.8	12.0	20.2	43.5	9.1	6.4
	T∩	6.8	17.2	25.2	24.6	20.3	5.8
	TΜ	12.5	26.6	18.3	10.2	26.5	5.8

#### Test plot design

The test plot (10 × 10 m) in the study area in 2021 was selected on flat terrain with a typical vegetation distribution and enclosed with fences to prevent interference from humans and livestock. A drought shed (8 × 8 m) was set up in the center of the test plot before May to intercept natural precipitation, and all simulated precipitation was artificially produced.

The experimental plot adopted a random block design with four replicates for each precipitation distribution pattern for a total of 16 plots. Each plot area measured 1 × 1 m, and an interval of 0.5 m was set between plots. A 40-cm-deep PVC plate was buried around each plot to prevent horizontal migration of soil moisture, and a space of 5 cm was reserved above the ground to prevent interference from surface runoff.

#### Manipulative experiment

The layout of the test plot was completed before May 2021, and a vegetation survey of the test plot was carried out on May 1 to determine the initial values of the vegetation index. Then, the timing of artificial precipitation was set according to the schedule listed in Table 2. For example, in the TΛ7 treatment, there were four precipitation events in May, with each comprising 5.1 mm. Artificial precipitation was performed on May 1, May 8, May 16, and May 24. Vegetation surveys were conducted at the end of each month.

### Measurement methods

#### Plant community composition

Vegetation surveys were conducted at the beginning of the experiment (May 1) and the end of each month from May to October. The species, number, height, frequency, and coverage were recorded. To reduce errors caused by human evaluation, a quadrat with the same area as the plot (1 × 1 m) was divided into 100 small squares measuring 10 × 10 cm. The cumulative number of squares occupied by each plant was regarded as the frequency of each species; the sum of the number of plants/clusters in 100 small squares of each plant was regarded as the density of each species, and the percentage of the vertically projected area of each plant in the quadrat was regarded as the coverage of each species. Owing to the sparse vegetation in the desert steppe ecosystem, the degree of overlap in coverage between species was negligible.

#### Plant community diversity

The Species richness, Pielou, Shannon–Wiener, and Margalef indices were used to evaluate species diversity in the test plot [40].

Species richness was defined as the number of species present in a 1 × 1 m quadrat.

The Pielou index is defined as follows:

E=Hlns (1)

The Shannon–Wiener index is calculated as follows:

$$H=-\sum_{i=1}^{s}P_{i}\;\text{ln}\;P_{i}$$ (2)

The Margalef abundance index is defined as follows:

$$A=\frac{S-1}{\text{ln}\;N},$$ (3)

where S is the number of species in the community; N is the total number of individuals of all species; P_i_ is the ratio of species i to all species in the plot (P_i_ = N_i_ /N), and N_i_ is the number of individuals of species i.

#### ANPP

In this study, the plot size was 1 m^2^, and indirect coverage regression was used instead of mowing to obtain the ANPP and avoid clipping effects [3, 41]. When the plant community aboveground biomass peaked during late August, 20 correction plots (1 × 1 m) were randomly selected near the test plot. The same observational method was used to investigate the plant community composition in the test and correction plots, and then the plants in the correction plots were immediately mowed down, identified according to the species, sealed, and brought back to the laboratory, where they were heated at a temperature of 65°C until a constant dry weight was obtained (ANPP). Correction plot biomass-cover regressions were established, and estimates of the slope were used to transform the plant cover to the test plot value of ANPP.

According to the vegetation survey, plant species in the test plots were classified into four plant functional groups based on life forms [19], among which perennial grasses included *S*. *breviflora*, *C*. *songorica*, and *Cleistogenes squarrosa* (Trin.) Keng; annual forbs included *N*. *pectinata* and *Salsola collina* (Pall.) Akhani & Roalson; perennial forbs included *Medicago ruthenica* (L.) Trautv., *Convolvulus ammannii* Desr., *Artemisia scoparia* Waldst. & Kit., *Aster altaicus* Willd, *Lagochilus ilicifolius* Bunge, *Allium mongolicum* Regel and *Allium tenuissimum* L., and semi-shrubs included *Bassia prostrata* (L.) Beck and *A*. *frigida*. Correction plot biomass-cover regressions were established for each plant functional type at each site (S1 Fig in S1 File): perennial grass *ANPP* = 5.68 × *cover* + 0.22, *R*^2^ = 0.74, *P* < 0.001; annual forb *ANPP* = 19.73 × *cover* − 5.09, *R*^2^ = 0.92, *P* < 0.001; perennial forb *ANPP* = 4.68 × *cover* − 0.05, *R*^2^ = 0.40, *P* < 0.001; semi-shrub *ANPP* = 3.43 × *cover* − 0.06, *R*^2^ = 0.83, *P* < 0.001.

### Data processing and analysis

Statistical analyses were performed and figures and tables were constructed using SPSS 21.0 (SPSS Inc., Chicago, IL USA) and Microsoft Excel 2010. The effects of the coefficient of variation (CV) of the growing-season precipitation on the total and plant functional group ANPP were tested. The growing-season precipitation CV was defined as the ratio of the standard deviation (STD) of precipitation to the mean (*CV* = STD/Mean×100%). One-way analysis of variance (α = 0.05) was used to examine differences in diversity and ANPP among the four precipitation distribution patterns. The sum of the biomass of all species in the plots was defined as the total aboveground biomass.

## Results

**Fig 3:**
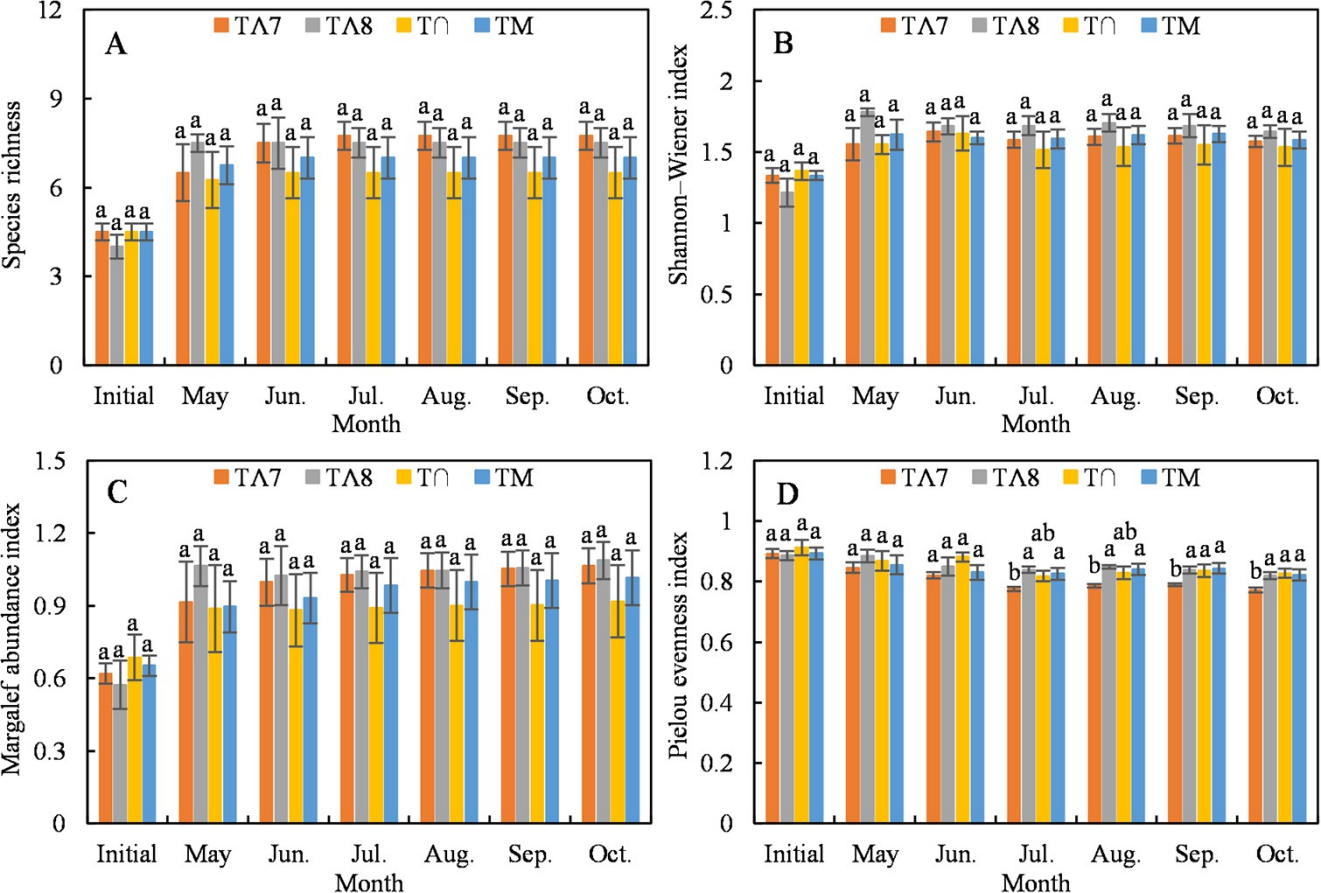
Temporal variation in plant community diversity for the four precipitation treatments during the growing season for (A) species richness, (B) the Shannon–Wiener diversity index, (C) the Margalef abundance index, and (D) Pielou evenness index. Bars indicate mean values (±SE, n = 4) for each treatment. Different letters indicate significant differences among treatments within a month (*P* < 0.05). The same letter indicates a nonsignificant difference.

The plant species, diversity, and abundance indices increased rapidly during May and then remained essentially unchanged during the remainder of the growing season. However, the distribution of precipitation had no significant effect on the *S*. *breviflora* desert steppe (*P* > 0.05, Fig 3A–3C). The effect of the precipitation distribution on the evenness index showed a significant difference after July (*P* < 0.05, Fig 3D).

**Fig 4:**
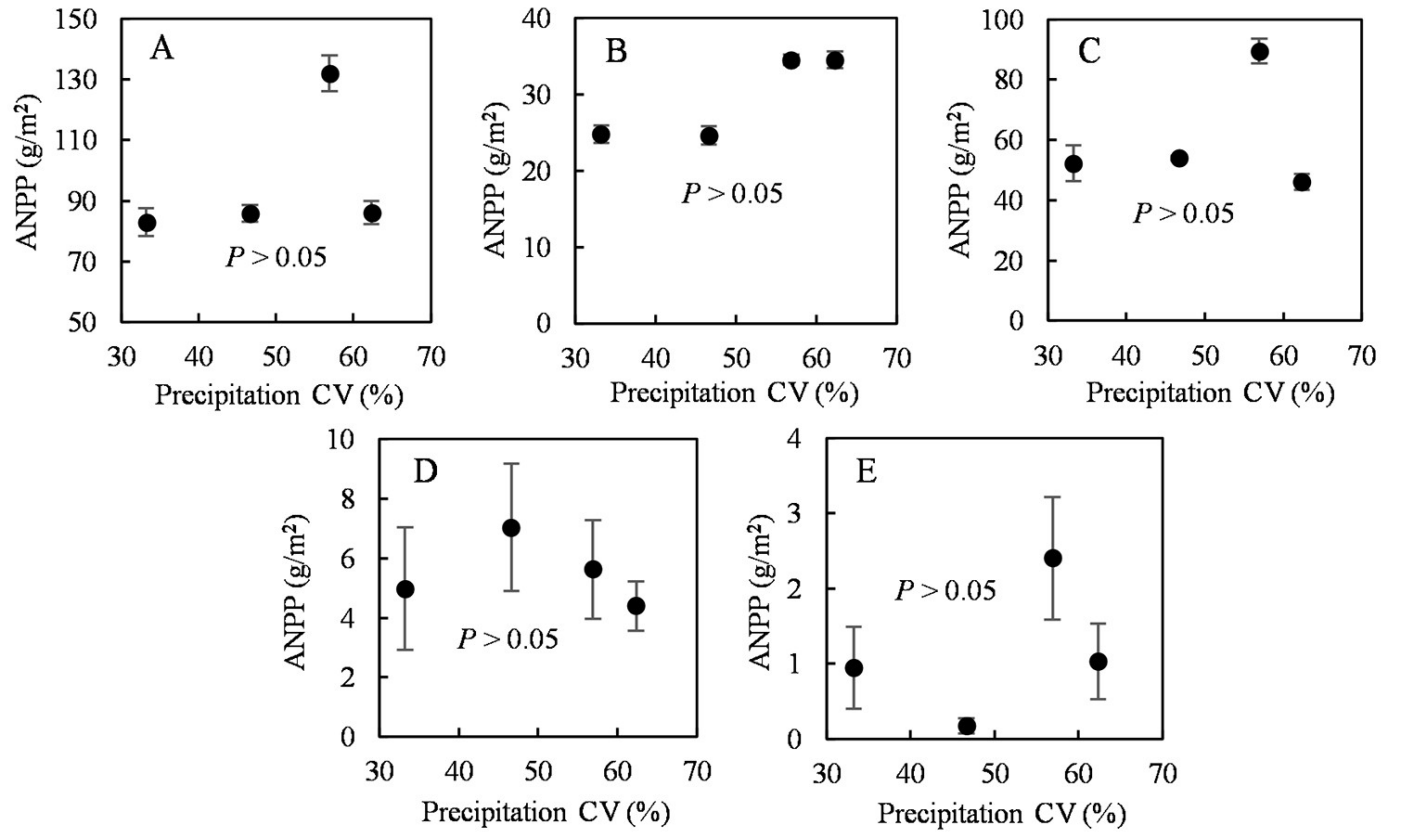
Effects of the coefficient of variation (CV) of the growing-season precipitation on functional ANPP groups. ANPP during September is shown as a function of the CV of precipitation for the ANPP of (A) total, (B) perennial grasses, (C) annual forbs, (D) perennial forbs, and (E) sub-shrubs. Points indicate mean values (±SE) for each treatment (n = 4).

The ANPP of the experimental plots was estimated according to ANPP allometric regressions for the four different plant functional groups (S1 Fig in S1 File). The nonsignificant linear, exponential, and quadratic relationships among ANPP in different plant functional groups and the coefficient of variation (CV) for precipitation (Fig 4) demonstrate that the growing-season precipitation CV did not significantly affect ANPP.

**Fig 5:**
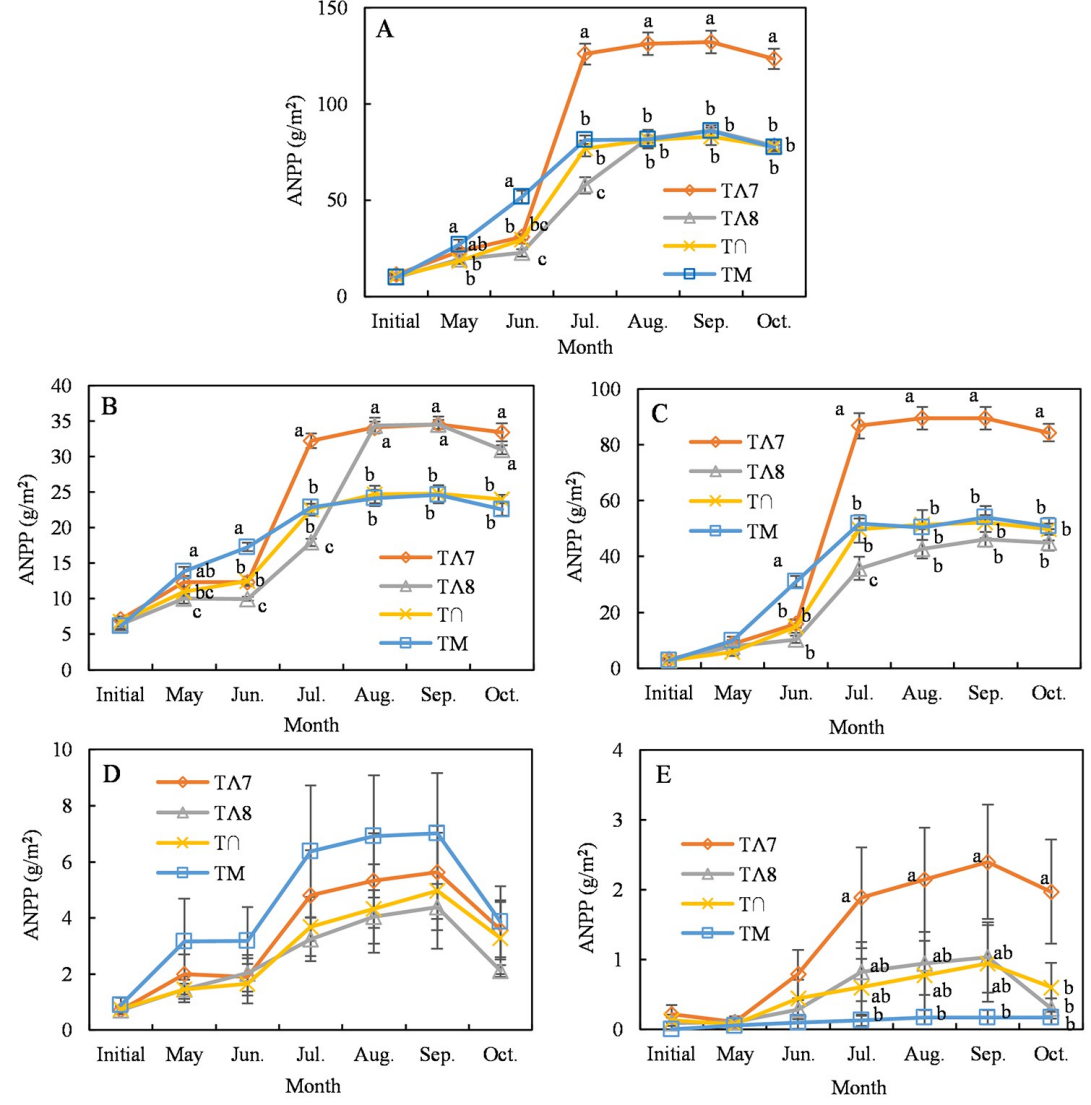
Effect of the distribution of precipitation on the vegetation community ANPP in the growing season for (A) total ANPP, (B) perennial grasses, (C) annual forbs, (D) perennial forbs, and (E) sub-shrubs. Symbols indicate mean values (±SE, n = 4) for each treatment. Different letters indicate significant differences among treatments within a month (*P* < 0.05). No letter indicates a nonsignificant difference.

The maximum total ANPP values all occurred during September; these were 132.09 g/m^2^ in TΛ7, 86.09 g/m^2^ in TΛ8, 83.03 g/m^2^ in T∩, and 85.81 g/m^2^ in TM. The TΛ7 ANPP was 55.4% higher than the mean total ANPP of the other treatments. The total ANPP was not significant before the experiment for the four precipitation distribution patterns. The total ANPP of TΛ7 was significantly higher than that of the other treatments from July to October. The total ANPP values of TΛ8, T∩, and TM were not significantly different from August to October (Fig 5A).

The perennial grasses ANPP of TΛ7 was significantly higher than that of the other treatments from July to October, and the maximum ANPP was 34.53 g/m^2^ during September (Fig 5B). The perennial grasses ANPP of TΛ8 was significantly higher than those of T∩ and TM from August to October and was not significantly different from that of TΛ7, and the maximum ANPP of TΛ8 was 34.54 g/m^2^ during September.

The annual forb ANPP of TΛ7 was significantly higher than that of the other treatments from July to October; the maximum ANPP was 89.54 g/m^2^ during September and accounted for 67.79% of the total ANPP (Fig 5C). The annual forb ANNP of TΛ8 showed no significant differences with T∩ or TM from August to October. The perennial forb ANPP showed no significant differences among the four precipitation treatments during the growing season (Fig 5D). The sub-shrub ANPP of TΛ7 was higher than that of the TM from July to October (Fig 5E).

**Fig 6:**
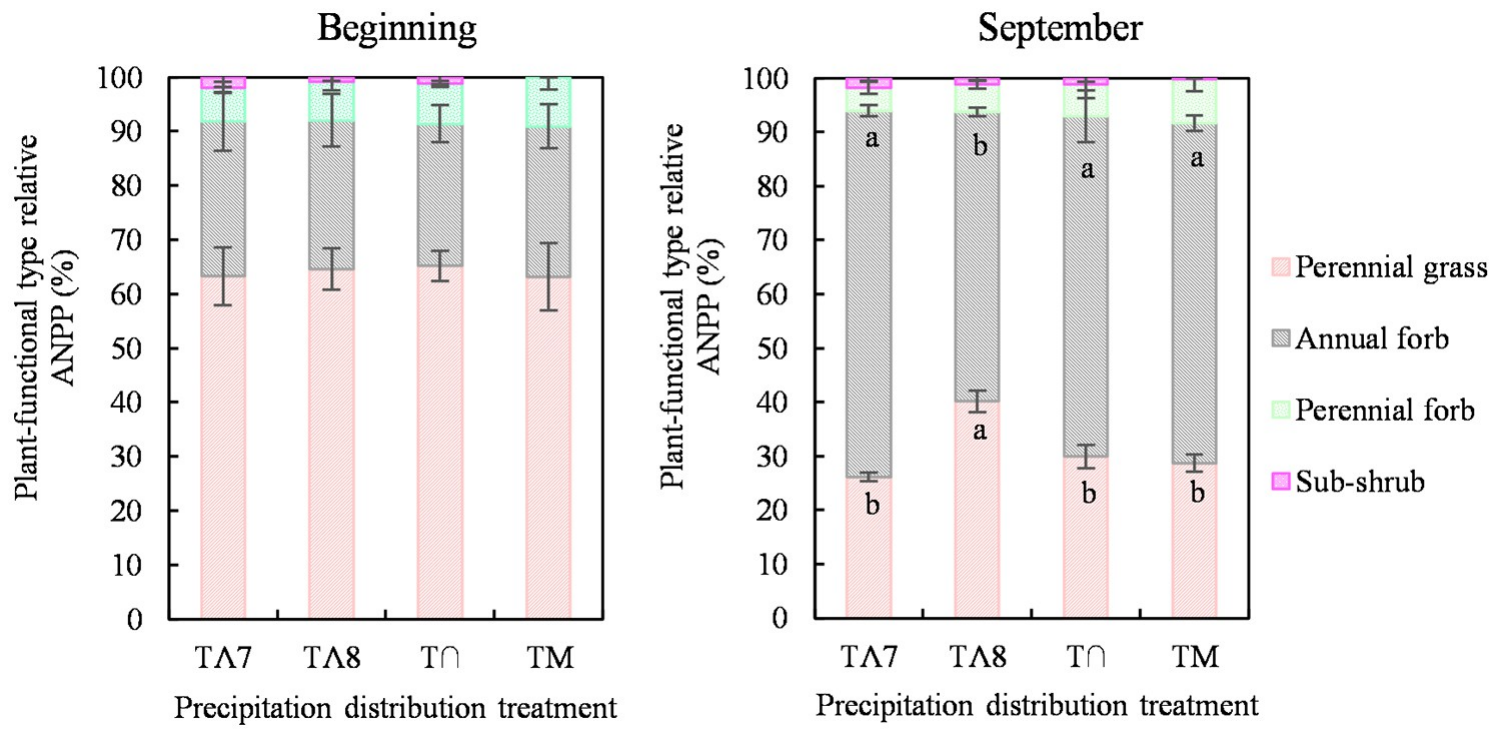
Plant-functional type relative ANPP at the beginning of the experiment and in September for the four precipitation distribution treatments. The symbols indicate mean values (±SE, n = 4) for each treatment. Different letters indicate significant differences among treatments within a month (*P* < 0.05). No letter indicates a nonsignificant difference.

The relative ANPP of both perennial grasses and annual forbs was > 90% in all four precipitation treatments at the beginning of the experiment and in September (Fig 6). The relative ANPP of the same plant-functional type among the four precipitation distribution plots showed nonsignificant difference at the beginning of the experiment. Perennial grasses were the main contributors, accounting for 64.1%, followed by annual forbs, accounting for 27.5%. However, the relative ANPP of the same plant-functional type showed significant differences between TΛ8 and the other three treatments in September. Annual forbs became the main contributors, accounting for 61.8%, followed by perennial grasses, accounting for 31.2%. The relative ANPP of perennial grasses in TΛ8 was higher than that in the other treatments, while the relative ANPP of annual forbs was less than that in the other treatments.

**Fig 7:**
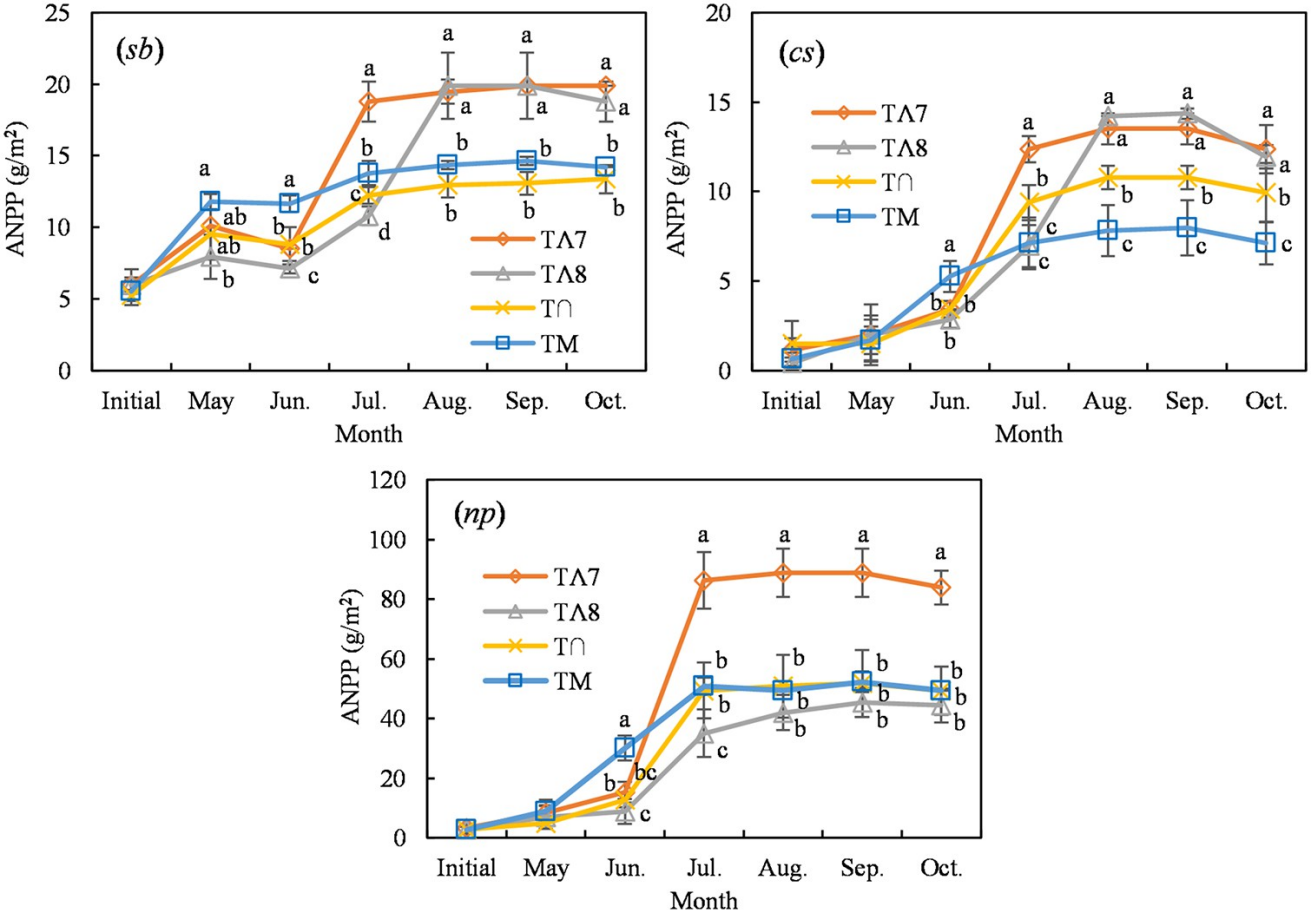
Effect of the distribution of precipitation on the ANPP of different vegetation types during the growing season for *S*. *breviflora* (*sb*), *C*. *songoric* (*cs*), and *N*. *pectinat* (*np*). The symbols indicate mean values (±SE, n = 4) for each treatment. Different letters indicate significant differences among treatments within a month (*P* < 0.05). No letter indicates a nonsignificant difference.

The ANPP values for *S*. *breviflora* and *C*. *songorica* in TΛ7 were significantly higher than those of the other treatments from July to October; the maximum ANPP values were 19.90 and 13.50 g/m^2^, respectively, during September (Fig 7). The *S*. *breviflora* and *C*. *songorica* ANPP values of TΛ8 were significantly higher than those of the T∩ and TM from August to October and showed no significant differences from that of TΛ7. The maximum ANPP values were 19.90 and 14.35 g/m^2^, respectively, during September. The ANPP of *N*. *pectinate* in TΛ7 was higher than that of the other treatments from July to October (Fig 7); the maximum ANPP was 88.80 g/m^2^ during September and accounted for 99.17% of the annual forb ANPP in TΛ7. The influence of the precipitation distribution on other vegetation ANPPs was not significant (S2 Fig in S1 File).

## Discussion

### The effect of the precipitation distribution on biodiversity

Precipitation is the primary factor limiting plant growth [42, 43] and is a predominant climatic factor for productivity and biodiversity in Inner Mongolian grasslands [19]. Soil water availability dominates the responses of the structure and composition of the plant community to climate in semiarid steppes [44]. However, our manipulation experiment did not provide sufficient evidence to support the first hypothesis. The change in the distribution pattern of precipitation during the growing season resulted in nonsignificant differences in plant species and diversity. Only the Pielou evenness index after July showed a significant decrease (Fig 3). The effects of variation in precipitation on plant functional group ANPPs were also nonsignificant (Fig 4). This may have been because the individual amounts of precipitation in the manipulation experiment were the monthly averages divided by the frequency (4–6 times per month), and this weakened the magnitude of variation in precipitation and prevented extreme precipitation and persistent drought events (Table 2). Such events cause temporal variability in available soil moisture, a factor that largely determines plant diversity and ecosystem functioning [3]. Knapp et al. (2002) found that increased precipitation variability during the growing season increased plant species diversity and reduced ANPP, independent of the changes in total precipitation. In our study, even moderate precipitation (4–20 mm) and intense evaporation may have only moistened the upper soil layers and transferred little water to the deeper layers, and thus could only be used by grasses with relatively shallow roots [45] and thus was not conducive to the growth of deep-rooted plants [46]. Specifically, perennial grasses and annual forbs accounted for > 90% of the total ANPP in our study (Fig 6), comprising *S*. *breviflora* (a C3 grass), *C*. *songorica* (a C4 grass), and *N*. *pectinate* (a C3 grass) (Fig 7). These species primarily use soil water at a depth of 0–30 cm [47]. Moreover, Knapp et al. (2002) found that increased variability in precipitation had a direct and strong effect on productivity by reducing the midsummer net photosynthesis of C4 grasses during the critical vegetation harvest period of the growing season. However, C4 grasses had less ANPP, and C3 grasses had the highest ANPP in our study.

It is also possible that the experiment was conducted over only one growing season, all the plots had the same environmental factors before the manipulation experiment in our study, and this led to the lack of an effect of the distribution of precipitation on plant diversity. Precipitation from the previous growing season (both seasonal and total precipitation) can alter the abundances of functional groups, and lagged precipitation effects are generally stronger for forbs than for grasses [4]. Thus, the effects of the precipitation pattern on plant diversity may be demonstrated in the coming years.

### Effect of the distribution of precipitation on ANPP

The total ANPP of TΛ7 was 55.4% higher than that of the other precipitation distributions (Fig 5A), supporting the second hypothesis. Moreover, the total ANPP of TΛ7 was 37.7% higher than the annual average ANPP (95.96 g/m^2^, 1961–2010) [48]. Our study was unique in that only the concentrated precipitation during July was critical for maximizing productivity on the desert steppe, whereas the differences among the other three precipitation distributions were not significant (Fig 5A). Guo et al. [1] and Song et al. [21] found that precipitation during July was the critical factor and had the highest correlation with vegetation fractional coverage on temperate grasslands in Inner Mongolia. The total maximum ANPP observed in TΛ7 was primarily caused by the rapid growth of the annual forb *N*. *pectinate* and the perennial grasses *S*. *breviflora* and *C*. *songorica* during July (Figs 5B and 5C and 7). The responses of the community and ecosystem to environmental change depend on the attributes of key species [49, 50]. The annual plants in desert ecosystems are special groups that are highly responsive to fluctuations in precipitation (Fig 6). The concentrated precipitation increases ANPP by stimulating a large number of annual plants to germinate, grow, and complete their life cycles over a short time [51]. With the increase in the precipitation gradient, the growth period, plant height, leaf length, and biomass of annual plants all increased. Moreover, the effective precipitation of 5 mm could allow plants to complete the life cycle, and the effective precipitation productivity of 70 mm allowed productivity to reach the maximum [52]. Moreover, the precipitation altered the height and leaf area of perennial grasses to boost ANPP [53]. Ye et al. [54] also found that the coverage and ANPP of *S*. *breviflora* were sensitive to precipitation. In addition, the air temperature reached its maximum during July (S3 Fig in S1 File), which, coupled with sufficient precipitation, encouraged rapid plant growth and ultimately maximum productivity. As a result, the TΛ8 (concentrated precipitation during August) did not result in attaining the total maximum ANPP.

The concentrated precipitation during June (TM) only resulted in the maximum ANPP during this month, rather than the greater annual ANPP (Fig 5A). This is because the precipitation was low during July (< 10 mm) and ineffective during August (< 5 mm); this may have caused a large number of annual plants to die without new seed germination [51]. The experimental site of this study was located in a typical desert steppe with low vegetation coverage, strong soil evaporation, sandy soil, and a low water-holding capacity; thus, rainwater evaporates quickly after infiltrating the soil. Annual plants are highly sensitive to rainfall and grow rapidly after rainfall during June; however, there was little precipitation during the critical period for biomass accumulation during July, and this prevented plant growth.

There was a significant positive correlation between annual precipitation and vegetation coverage when the annual precipitation was < 300 mm in the desert steppe, and the relationship weakened when the annual precipitation was > 300 mm [21]. This indicates that 300 mm of precipitation in the desert steppe can satisfy the requirements for vegetation growth. The synchronous variation in precipitation and air temperature (T∩) during the growing season did not result in the maximum annual ANPP, because although low but uniform precipitation results in a high annual vegetation germination rate, intense intraspecific competition leads to high plant mortality [51]. The annual precipitation (275.8 mm) at our study site was lower than the water requirement for plant growth, and the normal monthly precipitation caused the plants to remain in a state of water shortage, greatly increasing intraspecific competition.

In addition, it is noteworthy that the concentrated precipitation during July or August maximized the ANPP of the perennial grasses *S*. *breviflora* and *C*. *songorica*, and the values were not significantly different (Figs 5B and 7). This is because *S*. *breviflora* and *C*. *songorica* are characterized by the S strategy characterized by strong adaptability to extreme drought and stressful environments [55]. Moreover, *Stipa* has a high utilization efficiency in 0–30-cm soil water, which makes it highly adaptable to arid environments [47].

## Conclusions and summary

The influence of the distribution of annual precipitation on ecosystems is no less than that of interannual precipitation variation in grasslands. In this study, the effects of four major precipitation patterns during the growing season on the plant community in a desert steppe were investigated. We concluded that because of the adaptability of desert steppe vegetation to resource-poor environments, the variation among distributions of precipitation during the growing season had little effect on the vegetation community structure or composition but significantly affected aboveground biomass independent of total annual precipitation. The precipitation during July reached 41.6% of the annual average, and the precipitation during other months satisfied basic plant growth requirements; the desert steppe annual ANPP increased by 55.4%, primarily in the annual forb *N*. *pectinate*. Therefore, desert steppe productivity can be predicted several months in advance according to the precipitation during July, and management measures such as irrigation or artificial precipitation can be implemented during July to ensure maximum forage yield.

The results of this study indicate that the productivity of desert steppe is sensitive to the pattern of precipitation during the growing season, and the concentrated precipitation in July has the most significant influence on annual productivity. The results are based on the analysis of one year’s artificial rainfall experiment data, and there are thus several limitations and uncertainties. The amounts of artificial precipitation in each month were obtained by averaging the precipitation over the past 60 years; this may have diminished the variation in precipitation and excluded the influence of extreme rainfall. In addition, the results of this study are based on the data of the first year of the experiment, and the effects of the total precipitation of the previous year and the winter precipitation of the grassland ecosystem on the vegetation community in the second year cannot be ignored. This will be considered in future studies.

## Supporting information

S1 File (DOCX)

S1 Graphical abstract (PDF)
