# Supplementary material for: Precipitation in July maximizes total above-ground productivity of the desert steppe in Inner Mongolia, China
Source: PLoS One. 2024 Dec 16;19(12):e0314983. doi: 10.1371/journal.pone.0314983 (PMC11649078; doi:10.1371/journal.pone.0314983)
Supplement: S1 File — (DOCX) [file pone.0314983.s001.docx]

**S1 Fig. The ANPP allometric regressions for four different plant-functional types. Plant species ANPP as a function of cover at growth boom (at the end of August) for four functional types.**

**S2 Fig. Precipitation distribution effect on different vegetation type ANPPs during the growing season for: (*cs*) *Cleistogenes squarrosa*, (*sc*) *Salsola collina,* (*kp*) *Kochia prostrata* and (*af*) *Artemisia frigida*.; Symbols indicate mean values (±SE, n = 4) for each treatment. Different letters indicate significant differences among treatments within a month. No letter indicates a nonsignificant difference.**

**S3 Fig. The temperature variation of four precipitation distributions in growing season.**
