# Supplementary material for: Precipitation in July maximizes total above-ground productivity of the desert steppe in Inner Mongolia, China
Source: PLoS One. 2024 Dec 16;19(12):e0314983. doi: 10.1371/journal.pone.0314983 (PMC11649078; doi:10.1371/journal.pone.0314983)
Supplement: S1 Graphical abstract — (PDF) [file pone.0314983.s002.pdf]

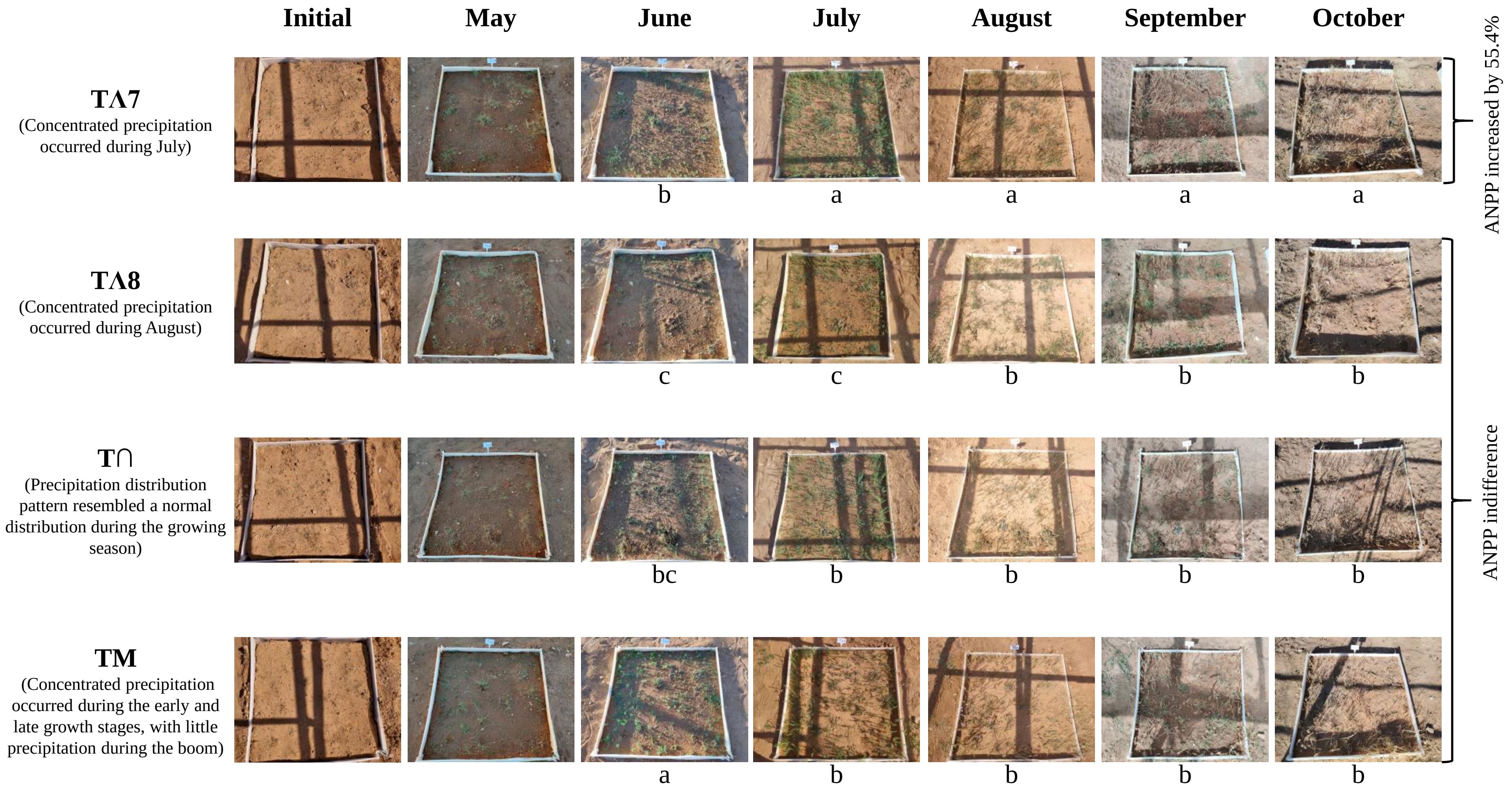

ANPP: the total above-ground net primary productivity; Different letters indicate significant differences among treatments within a month. No letter indicates a nonsignificant difference.
